# Elevated accuracy in recognition of subliminal happy facial expressions in patients with panic disorder after psychotherapy

**DOI:** 10.3389/fpsyt.2024.1375751

**Published:** 2024-06-13

**Authors:** Zirong Qian, Yunbo Yang, Katharina Domschke, Alexander L. Gerlach, Alfons Hamm, Jan Richter, Martin J. Herrmann, Jürgen Deckert, Volker Arolt, Peter Zwanzger, Martin Lotze, Bettina Pfleiderer, Hans-Ulrich Wittchen, Thomas Lang, Andreas Ströhle, Carsten Konrad, Winfried Rief, Thomas Suslow, Andreas Jansen, Tilo Kircher, Benjamin Straube

**Affiliations:** ^1^ Department of Psychiatry and Psychotherapy & Marburg Center for Mind, Brain and Behavior, Philipps-Universität Marburg, Marburg, Germany; ^2^ Department of Experimental Psychopathology, University of Hildesheim, Hildesheim, Germany; ^3^ Department of Psychiatry and Psychotherapy, Medical Center- University of Freiburg, Faculty of Medicine, University of Freiburg, Freiburg, Germany; ^4^ Clinical Psychology and Psychotherapy, University of Cologne, Cologne, Germany; ^5^ Department of Biological and Clinical Psychology, University of Greifswald, Greifswald, Germany; ^6^ Department of Psychiatry, Psychosomatics and Psychotherapy, Center of Mental Health, University Hospital of Würzburg, University of Würzburg, Würzburg, Germany; ^7^ Department of Psychiatry, University of Münster, Münster, Germany; ^8^ Functional Imaging Unit, Institute for Diagnostic Radiology and Neuroradiology, University of Greifswald, Greifswald, Germany; ^9^ Clinic for Radiology, University of Münster, Münster, Germany; ^10^ Institute of Clinical Psychology and Psychotherapy, Technische Universität Dresden, Dresden, Germany; ^11^ Department of Psychiatry and Psychotherapy, University Hospital, Ludwig Maximilians University, Munich, Germany; ^12^ Christoph-Dornier-Foundation for Clinical Psychology, Bremen, Germany; ^13^ Department of Psychiatry and Psychotherapy, University of Hamburg, Hamburg, Germany; ^14^ Constructor University Bremen School of Business, Social & Decision Sciences, Bremen, Germany; ^15^ Department of Psychiatry and Psychotherapy, Campus Charité Mitte, Charité - Universitätsmedizin Berlin, Berlin, Germany; ^16^ Department of Psychiatry, Agaplesion Diakonieklinikum Rotenburg (Wümme), Rotenburg, Germany; ^17^ Clinical Psychology and Psychotherapy, Philipps‐Universität Marburg, Marburg, Germany; ^18^ Department of Psychosomatic Medicine and Psychotherapy, University of Leipzig Medical Center, Leipzig, Germany; ^19^ Core-Facility Brainimaging, Faculty of Medicine, Philipps‐Universität Marburg, Marburg, Germany

**Keywords:** panic disorder, subliminal perception, facial recognition, forced choice, cognitive behavior therapy

## Abstract

**Background:**

Individuals with anxiety disorders (ADs) often display hypervigilance to threat information, although this response may be less pronounced following psychotherapy. This study aims to investigate the unconscious recognition performance of facial expressions in patients with panic disorder (PD) post-treatment, shedding light on alterations in their emotional processing biases.

**Methods:**

Patients with PD (n=34) after (exposure-based) cognitive behavior therapy and healthy controls (n=43) performed a subliminal affective recognition task. Emotional facial expressions (fearful, happy, or mirrored) were displayed for 33 ms and backwardly masked by a neutral face. Participants completed a forced choice task to discriminate the briefly presented facial stimulus and an uncovered condition where only the neutral mask was shown. We conducted a secondary analysis to compare groups based on their four possible response types under the four stimulus conditions and examined the correlation of the false alarm rate for fear responses to non-fearful (happy, mirrored, and uncovered) stimuli with clinical anxiety symptoms.

**Results:**

The patient group showed a unique selection pattern in response to happy expressions, with significantly more correct “happy” responses compared to controls. Additionally, lower severity of anxiety symptoms after psychotherapy was associated with a decreased false fear response rate with non-threat presentations.

**Conclusion:**

These data suggest that patients with PD exhibited a “happy-face recognition advantage” after psychotherapy. Less symptoms after treatment were related to a reduced fear bias. Thus, a differential facial emotion detection task could be a suitable tool to monitor response patterns and biases in individuals with ADs in the context of psychotherapy.

## 1 Introduction

Empirical studies have revealed that individuals with anxiety disorders (ADs) tend to prioritize the processing of threat-relevant stimuli in emotional information processing. This general cognitive framework is rooted in their anxious schemata (1), leading vulnerable individuals to exhibit increased susceptibility to subliminal-priming effects, hypervigilance towards fearful inputs, and a tendency to interpret emotional cues in a negative manner (2–6). Recognizing human facial expressions is crucial for interpersonal interactions (7), and the accuracy of identifying emotional facial presentation plays a vital role in facilitating appropriate activities and behaviour regulation (8, 9). The specific cognitive model of panic disorder (PD), a subtype of ADs, suggests that individuals with PD may misinterpret neutral faces as angry in facially expressed labelling test compared to general population (10).This misinterpretation could potentially result in dysfunctional responses in social situations (11).

Nevertheless, the notion of “universally acknowledged negative biases” remains somewhat contentious, as some studies have not observe recognition deficits in groups of patients with PD (12). For example, within the broader context of testing facial emotional recognition of basic emotions, PD patients showed no evidence of impaired recognition of disgust. In contrast, individuals with obsessive-compulsive symptoms (OCD) often perform worse at recognizing disgust (13). Corcoran et al. replicated these results, demonstrating that most participants with PD exhibited a similar recognition ability for disgust as healthy controls (14). These findings collectively indicate that the patients with PD may have a unique way of processing negative emotions. Moreover, many studies have posited that facial expressions can be rapidly recognized outside of conscious awareness, especially for threatening information, as they signal danger and increase the chances of survival (15, 16). However, the research has explicated that the tendency to misinterpret subliminally presented faces – those below the conscious threshold - as “angry” is notably heightened when these faces, serving as conditioned stimuli (CS+), have been previously paired with aversive outcomes (US) via a fear conditioning phase (CS-US association) (15). This underscores how pre-learned associations or successful conditioning can improve the likelihood of negative misinterpretations when identifying facial emotional cues at a pre-attentive level among patients with anxiety.

Cognitive behavior therapy (CBT), particularly exposure-based CBT, stands as the first-line approach for addressing ADs (17, 18). Exposure-based techniques within CBT primarily favor fear extinction learning, where individuals were systematically exposed to various CS+ in the absence of US. This exposure leads to a reevaluation of fear-related beliefs and avoidance behaviors (19–21). Consequently, people develop a secondary association between CS+ and US ( CS – no US association), resulting in augmented cognitive control over their vulnerability to negative cues. Neuroimaging evidence has unveiled a connection between the effects of psychotherapy and the normalization of brain activity in response to emotional facial stimuli. Specifically, during a facial emotion processing task, CBT has been associated with decreased activation in the amygdala and subgenual anterior cingulate cortex (ACC) when presenting both threatening and happy facial stimuli (22). On the other hand, patients with generalized anxiety disorder (GAD) who also have partial comorbidity with PD showed particularly heightened insula activity in response to happy stimuli (22). This stands in contrast to the blunted neural responses these patients exhibited in the amygdala and insula before undergoing psychotherapy (22). They suggest that CBT training may help elicit more neural responses towards positive stimuli, particularly in regions such as insula, known to be part of the human emotional brain (23, 24). Pillay et al. (2007) illuminates a “happy facial effect”, explaining that patients with PD might perceive happy cues as indicative of safety, thereby manifesting positive reinforcement or reassurance (25). More importantly, clinical training itself can be a positive and beneficial experience, one which perhaps enhances participants’ capacity for understanding emotional information and optimizing indices of optimism (26–28). In summary, we believe that undergoing CBT can help patients with PD move away from their usual “negative processing biases” allowing them to better manage their anxiety and become more receptive to positive signals.

Therefore, the primary objective of the current study is to investigate how individuals diagnosed with PD and treated with CBT identify facial emotional expressions when presented subliminally. To achieve this, we conducted an secondary analysis based on *PANIC-NET II* (29), a German national research network examining exposure-based CBT for PD. We utilized the subliminal affective recognition task, displaying faces briefly (happy, fearful, or mirrored) below the conscious threshold (33 ms), followed by backward masking with a neutral face to impede the detection of emotions on a conscious processing level (30). An uncovered condition was also included, where only the neutral mask was presented. Subsequently, a forced-choice task was administered in which participants recognized which face appeared or was thought to be seen (7). To evaluate the response patterns of patients with PD after treatment, we compared their response distribution of four types of responses between patients and healthy controls, and perform between-group comparisons for each type of stimulus: masked fearful, happy, mirrored, and uncovered conditions. Our initial hypothesis was that patients with PD were likely to show higher accuracy rates in the masked happy condition after CBT compared to healthy populations. Additionally, in the subliminal fearful, mirrored conditions, and uncovered condition, we anticipated only minor differences between the patients with PD and the healthy controls.

In addition to our main assumption about behavioral performance in the experiment, we also expected a potential correlation between residual anxiety symptoms and fear-related performance in patients. Given that state anxiety can affect the recognition of negative items (31, 32), our second hypothesis was that symptoms persisting after exposure-based CBT might moderate fear responses to non-fearful stimulus. More specifically, we suspected that individuals with lower anxiety-related symptoms might demonstrate reduced fear bias in the task. Thus, the false alarm rate for fear responses to happy, mirrored, and uncovered facial stimulus was considered an indicator of fear bias and was expected to correlate with patients’ clinical symptoms.

## 2 Methods

### 2.1 Participants and designs

Behavioral data were collected from patients with PD and healthy controls across sites in Münster, Greifswald, and Marburg (including patients from Würzburg), initially involving 40 patients and 50 controls. Specific inclusion and exclusion criteria are detailed in Yang et al. (2020) (29).

The current study used a between-group comparison, examining differences between treated patients who underwent exposure-based CBT (29), an effective intervention for ADs (33), and a group of healthy controls who did not receive any intervention. Pre-treatment data of this behavioural testing were not collected for either group to avoid repetition and training effects related to the stimuli and task procedures. To enhance data quality, individuals with consistent “uncover” or “mirror” responses or an accuracy rate below 10% in the uncovered condition were excluded. Consequently, 6 patients and 7 controls were removed from the analysis. This resulted in 77 participants (34 patients and 43 controls) for behavioral analysis.

Regarding the measurement of clinical symptoms, 29 patients provided data at two time points, allowing for correlation analysis both before and after treatment. These data give additional insights into the impact of CBT on clinical symptoms in patients with PD.

Detailed information regarding demographics and clinical aspects is displayed in **Table 1**.

**Table 1:** Description of demographics and clinical statistics.

Demographics		Patients (n=34)				Controls (n=43)			
Name	Category	N (%)	Mean	Median	SD	N (%)	Mean	Median	SD
**Age (years)**			30.09	28.00	9.40		33.00	29.00	10.33
**Gender**	**male**	14 (41.4)				12 (29.3)			
	**female**	19 (57.6)				29 (70.7)			
**Diagnosis**	**PD**	8 (24.2)							
	**PD/A**	25 (75.8)							
Symptom Scores		Patients (n=29)							
Name	Category	Mean	Median	SD	IQR	t/v	*p*	Effect Size	
**HAMA**	**baseline**	21.58		8.90		4.53	<.001	0.84	
	**post-treatment**	15.44		8.66					
**BDI**	**baseline**		13.79		8.15	6.74	<.05	0.51	
	**post-treatment**		11.31		9.23				
**ASI**	**baseline**	36.72		14.05		6.74	<.001	1.25	
	**post-treatment**	23.75		13.63					

PD, panic disorder without agoraphobia; PD/A, panic disorder with agoraphobia; HAMA, Hamilton Anxiety Rating Scale; BDI, Beck’s Depression Inventory; ASI, Anxiety Sensitivity Index. t is the statistical value for HAMA and ASI; v is the statistical value for BDI; for the Student t-test of HAMA and ASI, effect size is given by Cohen’s d; for the Wilcoxon signed-rank test of BDI, effect size is given by Rank-biserial correlation

### 2.2 Materials

**Figure 1:**
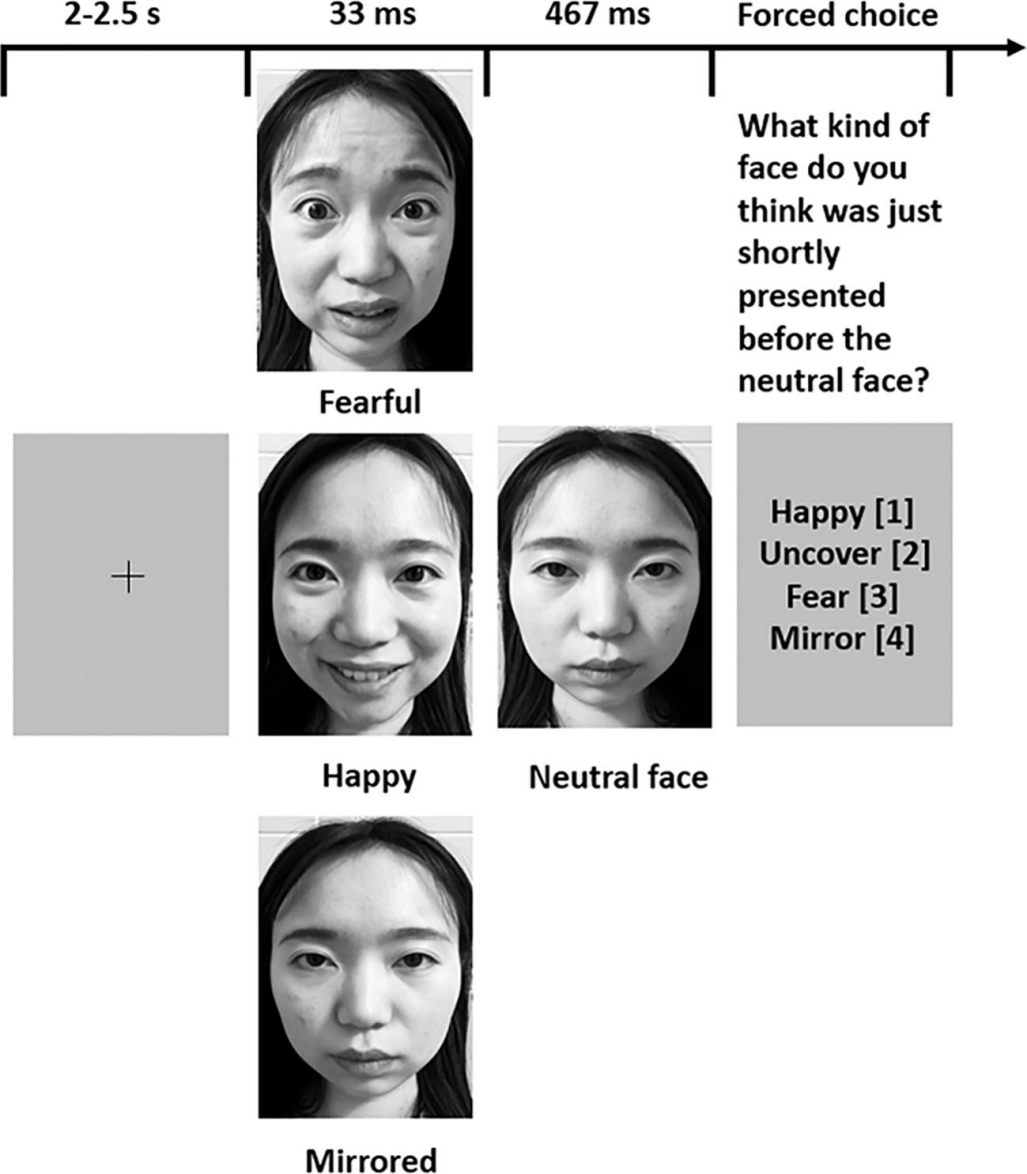
Subliminal affective recognition task procedure. There are four conditions in the task. Fearful (the fearful condition): a fearful facial stimulus is presented for 33 ms, followed by masking with a neutral face for 467 ms; Happy (the happy condition): a happy facial stimulus is presented for 33 ms, followed by masking with a neutral face for 467 ms; Mirrored (the mirrored condition): a mirrored face is displayed for 33 ms, followed by a rapid masking with a neutral face for 467ms; Uncovered (the uncovered condition): only the neutral face is presented for 500 ms. Following each trial, participants were asked to identify the face that had been masked from four possible choices, with the corresponding codes “happy”=[1], “uncover”=[2], “fear”=[3], or “mirror”=[4]. Their responses and reaction time for each trial were meticulously recorded. To illustrate the paradigm, we utilized comparable stimuli portraying facial expressions of the authors representing different emotions discussed in this article. The original stimulus materials can be provided upon request.

We adopted the subliminal affective recognition task to measure the detection of masked facial expressions. The stimulus set employed in the experiment is drawn from Ekman and Friesen (1976), known as Pictures of Facial Effect (POFA) (34). This facial expression database includes individuals from diverse ethnic backgrounds, encompassing both adult males and females, depicting six universally recognized emotions: anger, fear, sadness, happiness, disgust, and surprise (35). The procedure of our experiment is presented in **Figure 1**.

These questionnaires, including Hamilton Anxiety Rating Scale (HAMA) (36), Beck’s Depression Inventory (BDI) (37) and Anxiety Sensitivity Index (ASI) (38), were employed to measure the severity of anxiety and depression symptoms, and to identify specific anxiety-related concerns. Higher score on these scales indicates more severe clinical symptoms. These instruments have demonstrated high levels of validity and reliability, making them suitable for both academic research and clinical evaluation (36–38).

### 2.3 Analyses

First, we conducted an analysis of recognition performance by assessing the overall tendency within each condition, which involved aggregating counts of each response under distinct conditions in the task. To determine if there was a dependence between two nominal factors (Groups and Responses) in fearful, happy, mirrored, and uncovered conditions, we implemented the chi-square test of independence.

Second, fear bias, which is the false alarm rate for fear responses to happy, mirrored, and uncovered conditions, was calculated based on the frequency of selecting “fear” as the response in the non-fearful trials. The fear bias of patients then was correlated with the post-treatment scores of HAMA by using Spearman’s correlations, as these scores were not normally distributed.

Furthermore, we also undertook a series of exploratory analyses to examine the relationship between task-related performance and residual symptoms following treatment, along with clinical improvement. Specifically, we explored the correlation between fear bias and the Residual Gain Score (RGS) (39) of HAMA, BDI, and ASI, which indicates the extent of symptom relief after treatment. These correlations were evaluated using Spearman’s correlations. To address the concern of a potential negative selection bias, we defined “negative bias” as the proportion of “fear” responses out of the total reactions in the masked mirrored condition, which served as the control (or neutral) condition. We then examined the association between this negative bias with residual symptoms and both residual symptoms and clinical improvement using Spearman’s correlations. We analyzed the sensitivity (d-prime) for detecting fearful and happy signals, as well as the false alarm rate for happy responses in the task, following the principles of signal detection theory (SDT) (40). Details of these analyses are provided in the **Supplementary Materials** (see section 2-4, respectively).

These analyses were not pre-registered and involved secondary analyses.

## 3 Results

### 3.1 Response distribution

**Table 2** presents a response distribution of four distinct responses (happy, uncover, fear, and mirror) among two groups (Controls vs. Patients). The analysis is conducted separately for each condition. Each cell in the table represents the combined counts of response types along with their corresponding proportions. Detailed information regarding the total number of trials in each condition is reported in the section 1 of **Supplementary Materials**. The outcome of the chi-square test of independence demonstrates a dependence between the four responses and the two groups exclusively in the happy condition (χ^2^=17.9, *df*=3, *p*<.001). The p-value for the happy condition was corrected for multiple comparisons; *p*<.0125). This association is of relatively modest strength, characterized by Cramer’s V of.15.

**Table 2:** The combined distribution of four responses under four conditions respectively.

Conditions	Counts of Four Responses (Corresponding Ratio)								Statistical Results			*Cramer’s V*
	Controls (n=43)				Patients (n=34)				*χ^2^*	*df*	*p*	
	happy	uncover	fear	mirror	happy	uncover	fear	mirror				
**Fearful**	138 (32.1)	69 (16.1)	138 (32.1)	85 (19.8)	125 (36.8)	48 (14.1)	93 (27.4)	74 (21.8)	3.47	3	0.32	.06
**Happy**	187 (43.5)	68 (15.8)	91 (21.2)	84 (19.5)	193 (56.8)	28 (8.2)	68 (20.0)	51 (15.0)	17.90	3	<.001	.15
**Mirrored**	88 (19.9)	79 (17.9)	92 (20.8)	183 (41.4)	85 (23.5)	55 (15.2)	65 (18.0)	157 (43.4)	3.05	3	0.38	.06
**Uncovered**	8 (1.8)	401 (88.3)	9 (2.0)	36 (7.9)	5 (1.3)	337 (87.8)	4 (1.0)	38 (9.9)	2.39	3	0.50	.05

Each cell represents the aggregated absolute number of total responses from all participants within each group. The corresponding percentage of these combined responses relative to the total responses observed under each condition is provided in brackets.

Fearful (the fearful condition): the total responses are 430 for controls and 340 for patients, respectively; Happy (the happy condition): the total responses are 430 for controls and 340 for patients, respectively; Mirrored (the mirrored condition): the total responses are 442 for controls and 362 for patients, respectively; Uncovered (the uncovered condition): the total responses are 454 for controls and 384 for patients, respectively.

The p-values for the chi-square test of independence indicate a significant effect for the ‘Happy’ condition (corrected for multiple comparisons; p<.0125).

The *post-hoc* analysis explains that the categories of “happy” and “uncover” responses drove the group effect in the happy condition, with patients exhibiting a higher frequency of accurate “happy” responses (56.8% hit rate for the happy condition) compared to controls (43.5%). Consequently, we conclude that individuals with PD displayed a central tendency in the happy condition, showing a stronger inclination to select “happy” as their preferred choice compared to controls.

In the condition of uncovered, fearful and mirrored facial presentations, no significant group differences were observed. However, it is noteworthy that in the fearful condition, patients misclassified 37% of fearful faces as “happy,” while the control group marked 32% of responses as “happy” and 32% as “fear.”

### 3.2 Correlation analysis

The descriptive values for the false alarm rate in fear responses are reported in the section 3 of **Supplementary Materials**. In terms of clinical correlation, the results revealed a moderately positive correlation between the false alarm rate for fear responses for the happy, mirrored, and uncovered stimulus and post-treatment HAMA scores (r_s_=0.37, *p*<.05), indicating that lower post-treatment symptom scores were associated with a reduced fear bias in the task.

### 3.3 Exploratory analysis

As for the correlations between fear bias and clinical improvement, the fear bias and the RGS of ASI was found negatively correlated with each other (r_s_=0.43, *p*<.05), illustrating that patients who experienced a greater reduction in anxiety sensitivity following intervention tended to show less fear bias during the task. In relation to negative bias, positive correlations were observed with clinical symptoms at post-treatment of HAMA (r_s_ =0.37, *p*<.05), BDI (r_s_ =.59, *p*<.001) and ASI (r_s_ =.39, *p*<.05). Overall, these findings reflect that individuals with more residual anxiety and depression symptoms and higher anxiety sensitivity were more inclined to exhibit a negative selection tendency when recognizing mirrored or neutral facial presentations.

## 4 Discussion

Our study yielded two main conclusions. First, PD patients after CBT treatment showed a significantly better performance in detecting happy facial expressions than the healthy group. Second, patients with fewer anxious symptoms after psychotherapy demonstrated a reduced fear bias. Therefore, our assessment could serve as an effective instrument for tracking response patterns and biases in individuals with ADs in the context of psychotherapeutic interventions.

The accurate recognition rate for recognizing subliminal happy facial expressions revealed a “happy-face advantage” in post-treatment patients over controls, which is consistent with our first hypothesis. To the author’s knowledge, this paper is the first to report such phenomenon in the group of patients with PD who underwent exposure-based CBT in conducting subliminal affective facial recognition task. Correspondingly, positive detection tendency or emotional processing bias towards positive stimuli was also discovered by several studies but at subclinical levels among individuals with social-anxiety conditions, despite the widely acknowledged scientific consensus of a negative processing bias in this population (5). Regarding studies that consciously presented cues, Silvia et al. (2006) illustrated that both the high-anxiety social anxiety and low-anxiety social anxiety groups showed a significant recognition advantage for happy expressions over sad expressions. However, the high-anxiety group exhibited a longer response time to happy signals than those with low anxiety (41). Moreover, evidence of the highest hit rate (referring to the condition in which a signal is presented in tasks and participants provide correct responses according to signal detection theory) for happy emotions over negative emotions was also found in both low and high social anxiety participants (42). Considering documentation related to patients with PD, McNally et al. (1992) have posited that their participants rated positive words as more emotional than catastrophe words (43). Furthermore, gender difference also played a moderating role in attentional bias, with the highly anxious males exhibiting a bias towards happy expressions in the dot-probe task, whereas high-anxious females uncovered a bias towards angry faces (44). In the measurement of subliminal presented stimuli, similar to our study, when faces were displayed for 32 ms, Thomsen et al. (2011) observed healthy samples with greater accuracy in identifying happy faces compared to sad faces (45). Evidence from a comorbidity perspective has shown that clinical depressed patients reacted more quickly to happy faces than angry faces in subliminal regulation goal priming conditions in the study of Zhang et al (46), a finding that corresponds with our clinical results indicating a positive correlation between the residual depressive symptoms measured by BDI and negative bias toward the mirrored condition in the task (Result 3.3). They imply that the depressive symptoms might moderate their responding when processing certain emotional cues. According to the neuroimaging findings from a fMRI study that presented subliminal facial cues, it was detected that the masked happy facial expression drives a significantly greater neural activation related to anterior cingulate and amygdala than masked sad face among healthy female adults (47). Comparable to our other fMRI study’s behavioral results, stroke patients demonstrated a higher recognition rate for happy expressions compared to other negative expressions such as fear, anger, and disgust, even though their ability to recognize happy expressions was also compromised (48).Overall, our study contributes to this line of research by demonstrating that the “happy detection privilege” in patients after psychotherapy is even more pronounced compared to healthy individuals. Three primary factors contribute to this phenomenon. First, the salience of a smiling mouth, marked by distinct muscle movements, facilitates the easy recognition of happiness without requiring complex analysis (49, 50). Second, individuals after psychotherapy often undergo an improved mood. Beck’s cognitive schema, Bower’s network model, and the content-specificity hypothesis collectively suggest that individuals with anxiety tend to process information aligned with their emotional state or cognitive framework (51–55). Hence, as positive emotions become more dominant following psychotherapy, driven by a significant reduction in core symptoms from baseline (as shown in **Table 1**), our patients revealed less negative bias with mitigated anxiety and depressive symptoms after psychotherapy, along with decreased fear sensitivity (see Result 3.3). This shift in their psychological state supplied some nutrients for patients to prioritize and respond more sensitively to happy emotions after treatment, consistent with the mood-congruent theory.

The results of the between-group comparisons showed no significant differences in the subliminal fearful, mirrored and uncovered conditions in line with our hypothesis. However, in the fearful condition, patients misclassified 37% of fearful faces as “happy,” while the control group marked 32% of responses as “happy” and 32% as “fear.” This illustrated that our patients seemed inclined to respond positively (mostly marking as “happy”) in a more lenient manner when faced with uncertain or ambiguous stimuli. While we failed to establish a causal relationship between CBT-induced changes and detection performance, psychotherapy may still be responsible for this effect. This is supported by our results 3.3, which highlight a negative correlation between the clinical improvement of anxiety sensitivity and fear bias. Previous studies have also rationalized how attentional mediation exercises in psychotherapy can enhance positive attention orientation, expectancy, and optimism bias (56).

Concerning the clinical results related to our second hypothesis, the significant correlation between the false alarm rate for fear responses and the severity of anxiety suggests that such responses are primarily linked to abnormal cognitive processing mechanisms (57). More importantly, patients with attenuated symptoms were less likely to make fear-related errors in the task, which was in line with our expectation. These results paint a clearer picture of how the severity of patients’ symptoms is connected to their cognitive biases and task performance.

In conclusion, we demonstrated a positive response tendency among post-treatment patients supporting the idea that effective psychotherapy promotes preferential processing of positive-related information, thereby cultivating a positive outlook. While our experimental approach may offer limited assistance in elucidating the therapeutic effect on behavioral changes, the facial emotion detection task could be a suitable tool to monitor response patterns and biases in individuals with ADs in the context of psychotherapy.

## Data availability statement

The original contributions presented in the study are publicly available. This data can be found at https://doi.org/10.6084/m9.figshare.25895704.v1.

## Ethics statement

The studies involving humans were approved by Philipps-University Marburg (project no. 171/09). The studies were conducted in accordance with the local legislation and institutional requirements. The participants provided their written informed consent to participate in this study.

## Author contributions

ZQ: Conceptualization, Investigation, Methodology, Writing – original draft, Formal analysis. YY: Conceptualization, Methodology, Supervision, Writing – review & editing. KD: Writing – review & editing. AG: Writing – review & editing. AH: Writing – review & editing. JR: Writing – review & editing. MH: Writing – review & editing. JD: Writing – review & editing. VA: Writing – review & editing. PZ: Writing – review & editing. ML: Writing – review & editing. BP: Writing – review & editing. HW: Writing – review & editing. TL: Writing – review & editing. AS: Writing – review & editing. CK: Writing – review & editing. WR: Writing – review & editing. TS: Writing – review & editing. AJ: Writing – review & editing. TK: Writing – review & editing. BS: Investigation, Methodology, Project administration, Supervision, Writing – review & editing.
