# Supplementary material for: Elevated accuracy in recognition of subliminal happy facial expressions in patients with panic disorder after psychotherapy
Source: Front Psychiatry. 2024 Jun 13;15:1375751. doi: 10.3389/fpsyt.2024.1375751 (PMC11209896; doi:10.3389/fpsyt.2024.1375751)
Supplement: Supplementary file 1 [file DataSheet_1.pdf]

Supplement

1. The detailed information regarding the different versions of the subliminal affective recognition task

Due to the fact that the different versions of subliminal experiments were conducted at four centers, there was not a consistent number of responses for the mirrored and uncovered conditions per subject.

There are four different conditions were included in the task, with a total of 10 trials each for the fearful and happy conditions across all participants. As for the mirrored and uncovered conditions, 34 individuals (12 in the healthy group and 22 in the patient group) completed 11 trials of the mirrored condition and 12 trials of the uncovered condition, as a result of variations in the experiment versions used at different sites. For the remaining participants, they completed 10 trials. Therefore, the total number of trials for each condition is as follows:

The happy condition:
430 trials for controls (10 trials per 43 controls);
340 trials for patients (10 trials per 34 patients).

The fearful condition:
430 trials for controls (10 trials per 43 controls);
340 trials for patients (10 trials per 34 patients).

The mirrored condition:
132 trials for controls who completed 11 trials (11 trials per 12 controls) plus 310 trials for the remaining controls (10 trials per 31 controls);
242 trials for patients who completed 11 trials (11 trials per 22 patients) plus 120 trials for the remaining patients (10 trials per 12 patients).

The uncovered condition:

144 trials for controls who completed 12 trials (12 trials per 12 controls) plus 310 trials for the remaining controls (10 trials per 31 controls);

264 trials for patients who completed 12 trials (12 trials per 22 patients) plus 120 trials for the remaining patients (10 trials per 12 patients).

2. The definition of behavioural parameters in d prime analysis (SDT)

Calculations are based on Signal Detection Theory (SDT). The "hit rate" for "happy" is the percentage of "happy" responses in masked happy trials, while the "false alarm rate" for "happy" is the percentage of "happy" responses in masked fearful, mirror, and uncovered trials. Similarly, the "hit rate" for "fear" is the proportion of "fear" responses in masked fearful trials. The "false alarm rate" for “fear” is the proportion of "fear" responses in masked happy, mirror, and uncovered trials. The sensitivity of "happy" is the Z score of its hit rate for “happy” minus the Z score of its false alarm rate of “happy” responses. Likewise, the sensitivity of "fear" is adjusted by the Z score of its hit rate for “fear” minus the Z score of its false alarm rate of “fear” responses.

3. The comparison of SDT between two groups

Table 1 reports that the d prime of hit rate, false alarm rate and sensitivity associated with fearful and happy face expressions. When comparing the two groups, it's evident that the patient group exhibited a marginally higher hit rate for happy expressions in comparison to controls (W=551.000, p=.064). None of the types of sensitivity and false alarm rate for both signals demonstrated statistical significance, even if there appears to be a tendency among patients to display a relatively higher sensitivity and false alarm rate for happy expressions and lower sensitivity and false alarm rate of fear than controls.

**Dependent variables**	**Patients (n=34)**		**Controls (n=43)**		**Statistical Results**		
	**Mean/Median**	**S.D/IQR**	**Mean/Median**	**S.D/IQR**	**t(W)**	**df**	***p***
**Hit rate of fearful signals**	0.250	0.300	0.300	0.400	817.000	-	0.373

**Hit rate of happy signals**	0.600	0.475	0.500	0.600	551.000	-	0.064
**False alarm rate for fear responses**	0.117	0.148	0.133	0.139	779.500	-	0.622
**False alarm rate for happy responses**	0.191	0.145	0.167	0.148	614.000	-	0.231
**Sensitivity of fearful signal**	-0.017	1.032	0.014	1.286	0.114	75	0.909
**Sensitivity of happy signal**	0.130	0.773	-0.103	1.159	-1.007	75	0.319

Table 1. *The hit rate, false alarm rate and sensitivity of subliminal presented fearful and happy faces.*

Note*. p is significance of independent two sample t-test and Wilcoxon Singed Ranks test. Mean and S.D for sensitivity of happy and sensitivity of fear; Median and IQR for hit rate of fear and happy and false alarm of happy and fear face.*

4. The correlation analysis between SDT and clinical indicators

4.1 The correlation between the sensitivity of fearful signals and anxiety symptoms

There was no significant correlation found between the sensitivity of fear signal and the post-treatment scores of HAMA (r_s=0.342, p>.05) by Spearman's Correlations.

4.2 Clinical improvement from pre- to post-treatment

We examined clinical improvement or therapy effects by assessing the absolute difference between baseline and post-treatment scores. Additionally, these effects were indicated by the Residual Gain Score (RGS), a measure typically used to control for the initial differences in baseline scores when evaluating changes in target variables in repeated measures designs (Steketee, 1992). To do this, we first transformed baseline scores (Z1) and post-treatment scores (Z2) into Z-scores. In our study, our aim was to assess symptom reduction from pre- to post-treatment. Therefore, clinical change was calculated by multiplying pre-treatment scores by the correlation between pre-treatment scores and post-treatment scores (r12), and then subtracting the post-treatment scores. The formula is RGS = Z1r12 - Z2. A higher RGS value indicates greater symptom relief. Subsequently, we examined the associations between absolute changes, RGS, and task-related performance using Spearman's Correlations.

Table 2 presents the associations between two categories of clinical indicators (absolute difference scores and between baseline and post-treatment, RGS, and behavioral parameters (sensitivity for fear and happiness, and the false alarm rate for fear and happy responses). We found a negative correlation between the RGS of anxiety sensitivity and the false alarm rate for fear, suggesting that patients who experienced a greater reduction in anxiety sensitivity tended to exhibit less fear bias in the task (r_s = -.43, $p < .05$).

**Variable**	**1.**	**2.**	**3.**	**4.**	**5.**	**6.**	**7.**	**8.**	**9.**	**10.**
**Absolute different scores of:**										
**1.HAMA**							.01	.32	-.06	-.06
**2.BDI**							-.00	.06	-.17	.00
**3.ASI**							.08	.03	-.16	.00
**Residual gain scores of:**										
**4.HAMA**							-.12	.29	-.20	-.03
**5.BDI**							-.15	.02	-.25	.03
**6.ASI**							.08	.09	**-.43***	-.02
**Behavioural parameters**										
**7.sensitivity of fear**										
**8.sensitivity of happy**										
**9.false alarm rate for fear**										
**10. false alarm rate for happy**										

***Table 2**.* Clinical correlations between absolute change from baseline to posttreatment, RGS and task-related performance.
Note. **p*<.05. p values were not adjusted for multiple tests. N=29.

5. Alternative definition of positive and negative bias

5.1 The positive bias and negative bias towards mirrored stimuli between two groups

Since the mirrored condition consists of neutral faces, their responses as “happy” and “fear” were considered as their positive bias and negative bias towards subliminal neutral stimuli, respectively. The formulas for positive bias and negative bias are as follows, characterized by the frequency of “happy” and “fear” responses in total responses. Due to the non-normal distribution of their sample, we applied the Wilcoxon

rank test to assess group differences in positive bias and negative bias. The results revealed no significant differences in both positive bias and negative bias, although the patient group appeared to exhibit a slightly higher positive bias, while the healthy group had a somewhat more negative bias based on their median values (Positive bias: w=639.5, p>.05; Negative bias: w=830.5, p>.05).

$$Positivebias = \frac{Counts\ of\ response\ as\ happy(Count_{\mathrm{happy}})}{Counts\ of\ sum\ responses\ (Count_{sum})}\ [only\ in\ MN\ condition]$$

$$Negativebias = \frac{Counts\ of\ response\ as\ fear(Count_{\mathrm{fear}})}{Counts\ of\ sum\ responses\ (Count_{sum})}\ [only\ in\ MN\ condition]$$

Items	Controls		Patients		W	*p*
	Median	IQR	Median	IQR		
**Positive bias**	0.182	0.209	0.191	0.200	639.500	.403
**Negative bias**	0.200	0.200	0.100	0.273	830.500	.305

Table 3. The comparison about positive bias and negative bias between two groups.
Note. *p is significance of Wilcoxon rank test.*

5.2 The correlations analysis between post-treatment scores, treatment improvement and positive, negative bias

Clinical correlation between clinical parameters of post-treatment scores, treatment improvement (RGS) and positive or negative bias. Spearman's correlation test revealed that anxiety symptoms, depressive symptoms as well as anxiety sensitivity at post-treatment were positively correlated with negative bias (HAMA: r_s =.37, p<.05; BDI: r_s =.59, p<.001; ASI: r_s =.39, p<.05).

Variable	1.	2.	3.	4.	5.	6.	7.	8.
Post-treatment scores of								
1.HAMA							.05	**.37***
2.BDI							-.03	**.59*****
3.ASI							.024	**.39***
Residual gain scores of								
4.HAMA							-.03	-.10
5.BDI							-.05	-.08
6.ASI							.00	-.36

Behavioural parameters	
7.positivebias	
8.negativebias	

Table 4. Clinical correlations between post-treatment scores of HAMA, BDI, ASI or RGS of HAMA, BDI and ASI and positive or negative bias.
Note. **p*<.05, ***p*<.01, ****p*<.001. p values were not adjusted for multiple tests. N=29.

6. Analysis of Reaction time in task

6.1 *Table 5* indicated that the comparison of reaction time for each condition separately across two groups. As they did not meet the requirement of normal distribution for parametric test, Kruskal-Wallis test was applied to examine the median difference between four conditions. The results revealed that there was significant difference of reaction time with regards to different conditions ($X^2(3)$ = 165.9, *p*<.001).

Condition	N	Median	IQR	Statistical Results
**Fearful**	770	1.81	1.51	$X^2(3)$ = 165.9
**Happy**	770	1.76	1.61	*p* <.001
**Mirrored**	804	1.88	1.78	
**Uncovered**	838	1.31	1.06	

Table 5. The comparison between four conditions across groups. N is the total number of trials of each condition.
Note. *p is significance of Kruskal-Wallis test.*

6.2 The comparison of reaction time of two groups across four conditions. The results of Wilcoxon rank test concluded with reaction time of patient group was significantly faster than those with control group (w=6014720, p<.001).

Groups	N	Median	IQR	Statistical Results
**Controls**	1756	1.81	1.53	W= 6014720
**Patients**	1426	1.49	1.35	*p* <.001

Table 6. The comparison of reaction time between two groups across four conditions.
Note. *p is significance of Wilcoxon rank test.* N is the total number of trials of each group.

6.3 The group comparison of reaction time for each condition. *Table 3* showed they did not have similar patterns of reaction time for each condition. Patients always responded more faster than control group (Fearful: w=383260, *df*=1, *p*<.001; Happy: w=363810, *df*=1, *p*<.001; Mirrored: w=429760, *df*=1, *p*<.001; Neutral: w=322418, *df*=1, *p*<.01).

Conditions	Patients	Controls	Statistical Results

	Median	IQR	Median	IQR	*W*	*df*	*p*
**Fearful**	1.56	1.34	2.02	1.51	383260	1	<.001
**Happy**	1.59	1.38	1.93	1.62	363810	1	<.001
**Mirrored**	1.68	1.57	2.10	1.83	429760	1	<.001
**Uncovered**	1.16	1.06	1.46	0.98	322418	1	<.01

***Table** 7.* The comparison of reaction time between two groups for each condition.
Note. *p is significance of Wilcoxon rank test.*

6.4 *Table 8* and *Table 9* showed corresponding reaction time of four choices respectively under each condition for two groups. In terms of control group in *Table 8*, they made fastest respond with "uncover" in fearful condition ($X^2(3)$ = 21.192, *p*<.001), while "happy" was their quickest response in happy condition ($X^2(3)$ = 37.266, *p*<.001). Under the condition of mirrored condition, they respond with shortest reaction time of "uncover" whereas longest of "fear" ($X^2(3)$ = 15.039, *p*<.001). In uncovered condition, "uncover" was the fastest response ($X^2(3)$ = 25.866, *p*<.001). For patient group in *Table 9*, in fearful condition, they also made fastest response as "uncover" and longest response as "mirror" ($X^2(3)$ = 22.649, *p*<.001). As for condition of happy cues presented, "uncover" was their shortest response rather than "happy", which different from reaction pattern of control group ($X^2(3)$ = 25.292, *p*<.001). "Uncover" also was their fastest response in mirrored and uncovered conditions while "mirror" seems to be hardest selection with longest reaction time (Mirrored: $X^2(3)$ = 11.964, *p*<.001; Neutral: $X^2(3)$ = 43.122, *p*<.01).

**Conditions**	**Corresponding RT of responses Median (IQR)**				**Statistical Results**		
	**Happy**	**Uncover**	**Fear**	**Mirror**	X^2	*df*	*p*
**Fearful**	1.74 (1.37)	1.65 (1.11)	2.36 (1.45)	2.32 (1.52)	21.192	3	<.001
**Happy**	1.60 (1.16)	1.94 (1.51)	2.37 (1.40)	2.74 (2.41)	37.266	3	<.001
**Mirrored**	1.94 (1.74)	1.56 (1.60)	2.62 (2.15)	2.22 (1.78)	15.039	3	<.01
**Uncovered**	2.81 (1.67)	1.42 (1.11)	2.78 (1.17)	2.05 (1.75)	25.866	3	<.001

Table 8. Control group's corresponding reaction time of four choices respectively under each condition.
Note. *p is significance of Kruskal-Wallis test.*

Conditions	Corresponding RT of responses				Statistical Results		
	Happy	Uncover	Fear	Mirror	X^2	*df*	*p*
**Fearful**	1.56	1.12	1.77	1.83	22.649	3	<.001
	(1.40)	(0.86)	(1.32)	(1.52)			
**Happy**	1.44	0.86	1.82	2.00	25.295	3	<.001
	(1.03)	(1.55)	(2.04)	(1.44)			
**Mirrored**	1.65	1.19	1.78	1.79	11.964	3	<.01
	(1.73)	(1.12)	(1.64)	(1.32)			
**Uncovered**	1.63	1.08	1.69	2.22	43.122	3	<.01
	(1.04)	(2.56)	(0.69)	(1.69)			

Table 9. Patient group's corresponding reaction time of four choices respectively under each condition.
Note. *p is significance of Kruskal-Wallis test.*

6.5 The comparison between two groups regarding their reaction time of four choices across conditions. According to *Table 10,* we found patients group always get response faster than control group.

Responses	Corresponding RT of responses				Statistical Results	
	Controls		Patients		*W*	*p*
	Median	IQR	Median	IQR		
**Happy**	1.740	1.360	1.484	1.265	389101	<.001
**Uncover**	1.475	0.960	1.084	0.997	552008	<.05
**Fear**	2.415	1.553	1.784	1.582	229600	<.001
**Mirror**	2.323	1.971	1.876	1.485	353896	<.001

Table 10. The comparison between control and patient group.
Note. *p is significance of Wilcoxon rank test.*
